# Case report and literature review of gallbladder paraganglioma in patient with inflammatory bowel disease

**DOI:** 10.1093/jscr/rjaf426

**Published:** 2025-06-18

**Authors:** Matea Dominkovic, Lachlan Allan, Henry Pleass

**Affiliations:** Department of General Surgery, Westmead Hospital, Westmead 2145 Australia; Department of General Surgery, Westmead Hospital, Westmead 2145 Australia; Department of General Surgery, Westmead Hospital, Westmead 2145 Australia

**Keywords:** gallbladder, paraganglioma, inflammatory bowel disease, primary sclerosing cholangitis

## Abstract

Primary paraganglioma of the gallbladder is an extremely rare pathology. Only 21 cases have been reported in the literature, and we present the first reported case of gallbladder paraganglioma in a patient with inflammatory bowel disease and primary sclerosing cholangitis. A 54-year-old lady with history of chronic right upper quadrant pain on a background of inflammatory bowel disease and primary sclerosing cholangitis underwent laparoscopic cholecystectomy due to gallbladder polyps and gallbladder wall irregularity on surveillance imaging. Her histopathology incidentally noted gallbladder paraganglioma. Due to their scarcity, there is no consensus on management and follow-up of gallbladder paraganglioma. All previously reported cases have been thus far benign and adequately treated with cholecystectomy, however, intra-abdominal extra-adrenal paragangliomas have malignant and metastatic potential hence we would advocate for surveillance.

## Introduction

Paragangliomas are rare neuroendocrine neoplasms originating from the neural crest cells of the neuroectoderm [1]. Pheochromocytoma and carotid body tumors are the most common types of paragangliomas [2], however, primary paragangliomas can be found in any tissue except bone and lymph nodes [3]. All paragangliomas have malignant potential and therefore locoregional management options consist of observation, surgical resection, or radiation therapy [4].

Primary paraganglioma of the gallbladder is an extremely rare pathology, with only 21 cases reported in the literature [5]. Due to the challenging diagnosis, the majority of cases are diagnosed on histopathology following laparoscopic cholecystectomy [1]. The scarcity of the pathology and often retrospective diagnosis has not allowed management consensus within the literature or clinical practice guidelines to be formed [5]. Herein, we present a case of primary gallbladder paraganglioma in a patient with newly diagnosed inflammatory bowel disease (IBD) and primary sclerosing cholangitis (PSC), demonstrating the difficulties in diagnosis and an approach to long-term management. To the author’s knowledge, this is the first case published of gallbladder paraganglioma in a patient with IBD or PSC.

## Case

**Figure 1:**
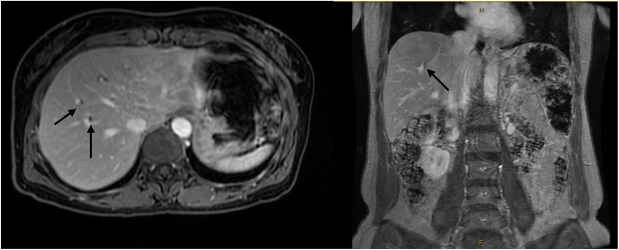
Magnetic resonance imaging liver/MRCP demonstrating subtle intrahepatic biliary dilatation (arrows), most pronounced in segment 8.

A 54-year-old female with a recent diagnosis of IBD complicated by PSC, presenting with right upper quadrant pain, underwent abdominal ultrasound, and magnetic resonance cholangiopancreatography (MRCP) as part of her PSC workup. Ultrasound demonstrated two 7 mm gallbladder polyps, with a normal gallbladder wall. MRCP demonstrated segmental intrahepatic duct dilatation in segments II and VIII in keeping with PSC (Fig. 1). Initial liver function tests and Ca 19.9 level showed only a mildly elevated GGT and ALP. Due to the recent diagnosis of IBD and small size of gallbladder polyps, observation with progress imaging at 12 months was undertaken. Repeat MRCP demonstrated a slight outpouching of the wall of the gallbladder with subtle enhancement of the lateral wall, with no concerning features for malignancy.

**Figure 2:**
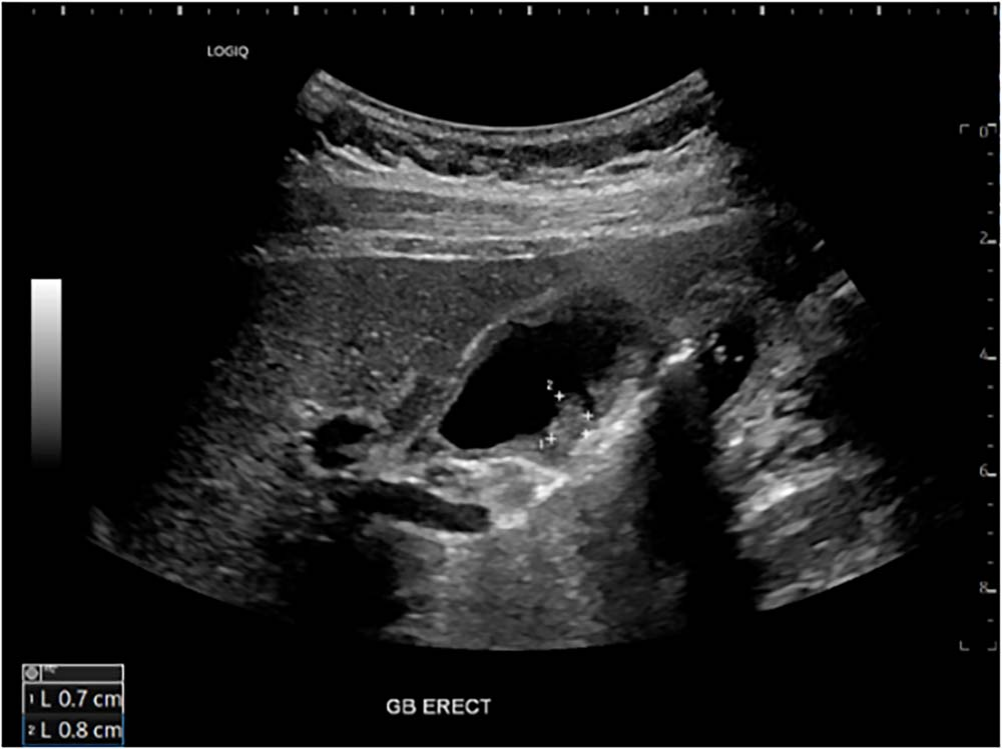
Preoperative ultrasound showing gallbladder polyp and wall thickening.

**Figure 3:**
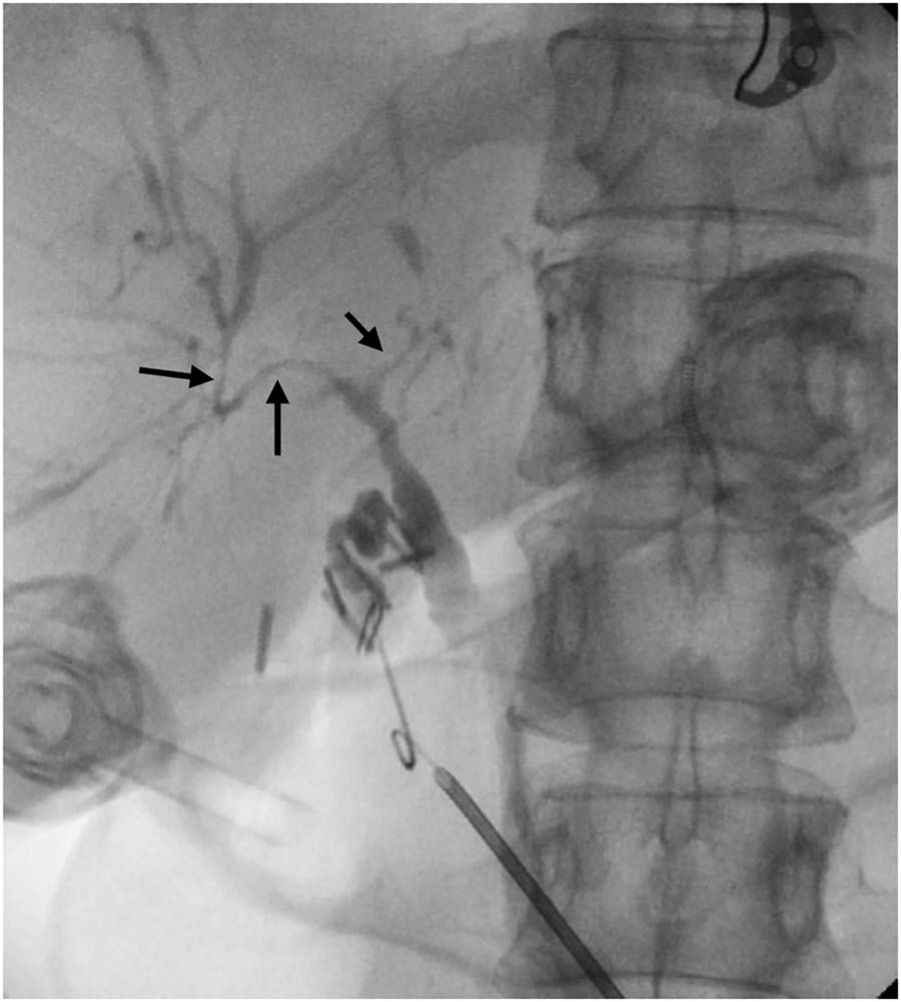
Intraoperative cholangiogram demonstrating intrahepatic duct stricturing (arrows) consistent with PSC.

Six months after surveillance imaging, she was presented with ongoing abdominal pain. Liver function tests (LFTs) and Ca 19–9 remained normal at this time. A repeat abdominal US demonstrated an irregular gallbladder wall with thickening up to 5 mm and two stable gallbladder polyps, the largest measuring 7 × 8 mm (Fig. 2). Due to ongoing symptoms and changes on imaging, a laparoscopic cholecystectomy with intraoperative cholangiogram was performed without complication. At the time of surgery, the gallbladder was noted to have an area of wall thickening thought to be a Phrygian cap. The operative cholangiogram was consistent with PSC, with narrowed intrahepatic ducts observed (Fig. 3).

**Figure 4:**
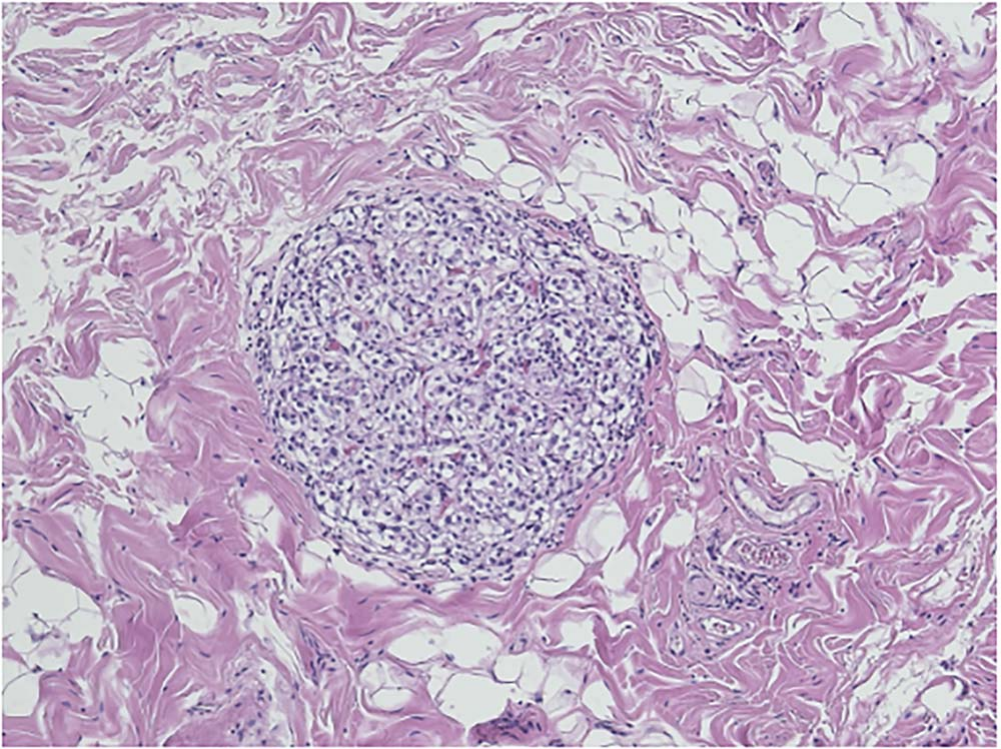
Gallbladder paraganglioma with characteristic Zellballen pattern seen in hematoxylin and eosin, ×20.

**Figure 5:**
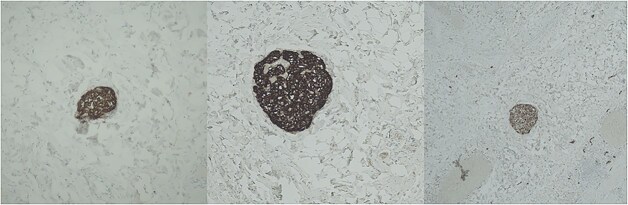
Immunohistochemistry for chromogranin (left), synaptophysin (middle), and S-100 (right).

Histopathological analysis of the gallbladder demonstrated follicular cholecystitis along with an incidental paraganglioma (Fig. 4). This lesion showed positivity with CD56, synaptophysin, and chromogranin staining with admixed S100-positive sustentacular cells (Fig. 5). Keratin was negative.

The case was discussed in the multidisciplinary team meeting, and surveillance with repeat CT imaging yearly for 5 years was planned.

## Discussion

The paraganglia consist of chief and sustentacular cells [6]. Together, the paraganglia form the paraganglionic system, which consists of the adrenal medulla and groups of extra-adrenal neuroendocrine cells [7]. When tumors arise from these cells, in the adrenal medulla, they are called pheochromocytoma, and in extra-adrenal locations, they are called paraganglioma [7]. Primary gallbladder paragangliomas are thought to arise from the hepatic plexus primordia that innervate the gallbladder [8]. Typically, extra-adrenal paragangliomas are found in the sympathetic plexus, commonly in pre-aortic and paravertebral areas [8]. Reports of paraganglioma of the gallbladder are exceedingly rare. Presentation is frequently vague with right upper quadrant abdominal pain and abnormal imaging. Most commonly diagnosis is made retrospectively on histopathological analysis.

Review of the literature reveals only 21 previous cases of gallbladder paraganglioma reported since the first case in 1972 by Miller *et al.* [9]. Gastroenteropancreatic neuroendocrine neoplasms have rarely been reported in association with IBDs [10]. Additionally, the prevalence of gallbladder abnormalities in patients with PSC, including gallstones, thickening of the gallbladder wall, and malignancy, has been reported to be 41% [11]. Of the few reported cases, biliary system paragangliomas are most commonly seen in 50–60-year-olds and are more common in female patients [7], with 6 males and 15 females reported. Some cases of paraganglioma can be hereditary, and some are familial and associated with other syndromes such as multiple endocrine neoplasia 2A or 2B, von Hippel–Lindau [12], or neurofibromatosis type 1 [13]. Current guidelines advocate for surgical resection for locoregional control of paragangliomas in patients with hereditary mutations [14]. Female gender, IBD, PSC, and certain familial syndromes could therefore represent risk factors for the development of gallbladder paraganglioma, but more studies are needed.

Up to 50% of intra-abdominal extra-adrenal paragangliomas have malignant potential [15], and 35%–40% of paragangliomas develop metastases [4]. Additionally, the local-regional recurrence rates of pheochromocytoma and paraganglioma after resection are reported to be 3%–16% [16]. Despite this, all prior reported cases of gallbladder paraganglioma have been benign and adequately treated with cholecystectomy [7]. However, due to their rarity, there are no guidelines for management or surveillance of gallbladder paraganglioma. Certain studies, therefore, recommend follow-up with Gallium-Dotatate PET [5]. In our institution, we advocate for surveillance with repeat CT yearly for 5 years due to the risk of recurrence of paragangliomas, however, more evidence and follow-up data is needed to establish guidelines for gallbladder paraganglioma.

## Conclusion

Paraganglioma of the gallbladder is an extremely rare condition which was malignant potential [4]. Due to the rarity of the condition, vague presentation, and often retrospective diagnosis, there is a paucity of literature to guide immediate and long-term management. We present the first reported case of gallbladder paraganglioma in a patient with IBD and PSC, demonstrating the complexities in diagnosis and management. Despite the malignant potential, cholecystectomy is likely adequate treatment; however, we recommend surveillance following resection.
